# Taste Plasticity in Nutrition and Health: A Scoping Review

**DOI:** 10.3390/nu17081336

**Published:** 2025-04-12

**Authors:** Hayeon Sung, Elizabeth Heaton, Monica Dus

**Affiliations:** Department of Molecular, Cellular, and Developmental Biology, The University of Michigan College of Literature, Science, and the Arts, Ann Arbor, MI 48103, USA; hayeons@umich.edu (H.S.); eheaton@umich.edu (E.H.)

**Keywords:** taste plasticity, dietary effects, nutrition, public health, obesity, GLP-1 agonists

## Abstract

Taste sensation is at the intersection of nutrition and health. Our food choices are shaped by the foods we like, but our dietary habits can, in turn, influence how we sense food. This phenomenon, known as diet-induced taste plasticity, has received renewed attention as a tool for designing healthier foods and retraining eating habits. This review synthesizes emerging data from humans and model organisms to characterize how dietary environments shape taste sensation while exploring its underlying molecular and physiological mechanisms. Although there is still so much to discover about this phenomenon, accumulating evidence suggests an inverse relationship between a tastant concentration and the ability of the organism to respond to it. The effects largely depend on diet, but body weight changes play a role in specific dietary settings. Several mechanisms are at play, including receptor abundance and function, neuroendocrine signaling, gene expression, and neuroinflammation. Many open questions on mechanisms remain, including the concentration and times of exposure for effects to manifest and the consequences for nutrition and health. Beyond mechanisms, future research should explore strategies to leverage taste adaptation for healthier food design.

## 1. Introduction

The chemosensory system of taste enables organisms to detect and differentiate chemical compounds in their environment, guiding dietary choices essential for survival. A key function of the gustatory system is to distinguish nutrient-rich foods from harmful substances. Far from being static, taste sensation is shaped by a variety of physiological and environmental factors, including metabolism, age, and disease [1,2,3,4,5].

Among these factors, diet-induced taste plasticity—the ability of the taste system to change with diverse dietary environments—is significant for nutrition and health, allowing organisms to efficiently use available food resources, especially when these become scarce. One striking example is found in insects that adapt to dietary deterrents. For instance, certain insects reared on a diet containing bitter plant-derived deterrents such as strychnine exhibit reduced responses to the bitter compounds and an increased acceptance of a bitter-containing diet [6,7,8,9,10]. A similar phenomenon has been observed in wild rodents, which respond differently to bitter compounds depending on their habitat [11].

While these adaptations to bitter compounds illustrate how dietary exposure can shape taste sensation in response to environmental constraints, a similar form of diet-induced taste plasticity occurs with macronutrient-rich foods, particularly those high in fat and sugar. Given the strong relationship between a high-calorie diet and weight gain, much of the research in this area has examined taste alterations in the context of diet-induced obesity. Obese individuals and animal models often exhibit blunted taste responses, particularly to sweet and fatty stimuli, and many studies have primarily examined taste alteration as a result of weight changes [12,13,14,15,16,17]. However, accumulating evidence suggests that dietary experience itself is also a driver of taste plasticity independently of weight changes. Studies in both animal models and humans indicate that exposure to diets high in fat and sugar reduces taste sensitivity, even in the absence of significant weight gain. For instance, chronic consumption of a high-fat or sugar diet leads to a diminished taste sensation, even in models where weight gain was genetically or pharmacologically inhibited [18,19,20].

Understanding how dietary environments shape taste and feeding behavior is key to designing interventions that encourage healthier eating habits and combat diet-related diseases. Food manufacturers could leverage the plasticity of the taste system to develop products that align sensory experiences with health goals. For instance, creating appealing foods with reduced sugar and salt content could help individuals “retrain” their taste preferences toward healthier choices. Public communication emphasizing the adaptability of the taste system could further empower individuals to adopt sustainable dietary habits.

This review synthesizes recent research from model organisms and humans on how dietary environments affect taste plasticity. It also explores open questions, including the molecular mechanisms driving taste plasticity, the interplay between inflammation and sensory adaptation, and the epigenetic regulation of the gustatory system.

## 2. Anatomy of Taste System

Taste is a critical sensory system that guides feeding behaviors in mammals and insects. Despite their evolutionary divergence, these two groups exhibit similarities in detecting and processing taste stimuli, even though their taste systems are structurally and molecularly distinct.

In mammals, taste is mediated by taste receptor cells (TRC), which continuously regenerate every 10–14 days [21,22]. These cells are housed within taste buds primarily located on the tongue. Taste buds are distributed among three main types of papillae: fungiform (anterior tongue), circumvallate (posterior tongue), and foliate (lateral tongue). These taste buds contain multiple TRCs that respond to distinct taste modalities—sweet, umami, bitter, sour, and salty—each activating specific intracellular signaling pathways. Sweet, umami, and bitter tastes are detected by G-protein coupled receptors (GPCR) of the T1R and T2R families, initiating intracellular signaling transduction by stimulating gustducin, which activates downstream cascades, including Phospholipase Cβ2 (PLCβ2) [23,24]. Sour taste is mediated by proton-sensitive channels, and salty taste involves epithelial sodium channels [24,25,26]. In addition to the five basic taste modalities, fat sensation is also recognized as a distinct taste modality, involving specific receptors that detect free fatty acids [18,27]. These signals are transmitted to the brain via afferent nerves, including the chorda tympani and glossopharyngeal nerves, which project to the nucleus of the solitary tract in the brainstem [22].

In contrast, insects rely on gustatory receptor neurons (GRNs), which are housed within sensilla found on multiple sites, including the proboscis, legs, and wings [28,29,30]. Unlike mammalian TRCs, which are renewed continuously, insect GRNs are terminally differentiated and do not undergo continual replacement. Insect taste receptors expressed in GRNs belong to a distinct family of ligand-gated ion channels, which directly generate electrical signals in response to tastants instead of relying on second-messenger cascades like mammalian GPCRs. These receptors form heteromeric complexes to detect sugars, bitter compounds, salts, and other chemical cues essential for feeding decisions [31,32,33,34]. GRNs project to the subesophageal zone (SEZ), the primary taste processing center in the brain [35,36].

## 3. Diet-Induced Taste Plasticity from Insects to Humans

Changes in taste sensation in response to dietary composition have been observed across various species, from invertebrates to mammals (Table 1).

In insects, much of this research has focused on how these animals adapt to unpalatable diets through sensory desensitization—a process that allows them to overcome the aversive taste of plant-derived deterrents and expand their feeding options.

A striking example comes from caterpillars of the moth genus *Helicoverpa*. When reared on a diet containing the bitter plant-derived toxin strychnine, these caterpillars displayed diminished responses in their taste sensilla to the compound [6,7]. Remarkably, they exhibited reduced neural responses and consumed strychnine-laced food at levels indistinguishable from caterpillars raised on a control diet. Research on *Drosophila melanogaster* further highlights the adaptability of insect taste system in aversive food environments. Flies fed camphor-laced diets exhibited reduced responses in bitter-sensing neurons and increased acceptance of camphor-containing foods [8]. These findings suggest that sensory systems can recalibrate when organisms repeatedly encounter specific compounds. However, this adaptability has limits. For more toxic compounds, such as quinine or strychnine, *D. melanogaster* flies did not show comparable desensitization, indicating that toxicity may constrain sensory plasticity. This limitation likely reflects an evolutionary balance, ensuring that while insects can adapt to exploit aversive food sources, their ability to avoid truly harmful toxins remains intact.

Indeed, caterpillars reared on tobacco exhibited a strong preference and high consumption of tobacco leaves, whereas those reared on cotton showed a greater inclination towards cotton leaves over tobacco leaves [57]. Similarly, caterpillars raised on a caffeinated diet ate caffeine-treated disks as readily as water-treated disks, adapting to the bitter compound [9]. Thus, adaptations to aversive tastants allow insects to adjust to challenging feeding conditions.

Taste plasticity can also manifest rapidly. For example, in *Manduca sexta* caterpillars, just 12 h of exposure to caffeine reduced neural responses and behavioral aversion to the compound [9,10]. This suggests that insects can adapt quickly to new chemical environments, potentially through receptor sensitivity shifts, alterations in signaling pathways, or changes in how the nervous system processes sensory information. This rapid plasticity may enable caterpillars to continue feeding on otherwise noxious plants while they learn about potential toxicity.

Chronic exposure to sweet compounds can also alter sweet taste sensation. Feeding *Drosophila melanogaster* flies a high-sugar diet (30% sucrose, fructose, or glucose) for more than five days led to reduced responses of sweet-sensing gustatory neurons in the labellar sensilla to sucrose [19,40,41]. This diminished neuronal sensitivity was mirrored behaviorally by lower proboscis extension responses to various sucrose concentrations and a deficit in locating sweet food sources [19,40]. Notably, our group found that these taste changes were independent of diet-induced obesity: genetically obese flies on a control diet had normal sucrose responses, while genetically obesity-resistant flies on a high-sugar diet showed reduced sucrose sensitivity [19]. Remarkably, the decrease in sweet sensation persisted for more than 20 days after reverting to a control diet through epigenetic mechanisms [41], suggesting that the neurons in the sensory system can remain locked into a past nutritional environment. These sensory alterations had profound implications for feeding behavior. Using neuro- and optogenetics, we demonstrated they directly and causally drove higher food intake and obesity by diminishing food reinforcement learning, primarily through lower stimulation of dopaminergic neurons and impaired dopamine release onto food memory circuits [58,59].

Artificial sweeteners can similarly reshape taste, eating, and metabolic responses. Wang et al. (2016) found that chronic consumption of sucralose in *Drosophila melanogaster* reprogrammed neural activity in sweet-sensing pathways, leading to higher feeding behavior and disrupted glucose homeostasis [37]. Dietary imbalances can also modulate taste sensitivity. Flies fed a sugar-enriched diet lacking yeast, their natural protein source, for four days exhibited reduced gustatory neuron responses to sucrose and shifted their feeding preference toward yeast extract rather than sugars like sucrose, fructose, and glucose [54]. In contrast, flies fed a sugar-reduced but yeast-enriched diet (no added glucose) showed heightened responses to sugars [54].

Together, these studies illustrate how diverse dietary experiences—whether bitter, high-sugar, artificially sweetened, or protein-deficient—alter the neural and behavioral components of taste sensation in insects. These findings also show that sensory changes have a direct and causal role in reshaping feeding behavior and metabolic health.

Dietary experiences also affect taste sensation in rodents. Blind mole rats (*Spalax galili*) exhibit population-level differences in bitter taste sensitivity that align with the soil types of their habitats. Those living in basalt soil—relatively less bitter yet rich in nutrients—displayed heightened sensitivity to bitter taste, presumably as a protective mechanism to avoid ingesting harmful substances in an otherwise resource-abundant environment [11,60]. In contrast, mole rats from chalk soil regions, where food is scarce, exhibited lower bitter responsiveness, increasing the range of acceptable food sources in a harsher habitat. Thus, similarly to insects, the modulation of bitter sensitivity is tightly linked to survival, enabling animals to balance the risks of toxin ingestion with the need to exploit limited food resources optimally.

Diets rich in fat or sugar also influence gustatory plasticity, often selectively. Rats with four-week access to 30% sugar water but standard chow exhibited attenuated chorda tympani nerve responses to sucrose applied to the tongue [43,44]. This attenuation was also observed with sodium chloride in females [43] but not in male rats [44]. Notably, these alterations to sucrose responses were present across concentrations without weight gain; however, this diet did not affect responses to glucose, fructose, maltose, sodium saccharin, NaCl, organic acids, or umami, indicating specific effects [44]. There was also no effect on the responses to mechanical or temperature stimuli [44]. Importantly, unlike in the fly, these deficits returned to normal after removing the sugar water for four weeks [44], which could be explained by the fundamental difference in the biology of taste in insects, where the taste receptor cells are neurons that do not renew [34].

Beyond affecting the sensory nerves, diet can also alter the responses of the taste cells and higher-order neurons involved in taste processing in the brain. Chronic consumption of a high-fat diet reduced the calcium responsiveness of isolated mouse taste cells to sweet stimuli like saccharin and acesulfame potassium and to the bitter compound denatonium; some of these deficits were dependent on diet, while others were also dependent on weight gain [16,20]. The high-fat diet mice showed lower preference for the sweet tastants at lower or higher concentrations, depending on the compound; for saccharin, the obese mice were less sensitive than controls at low concentrations. A high-energy diet (high in fat and carbohydrates) also decreased the magnitude and duration of taste responses in the nucleus of the solitary tract of obese rats. Interestingly, there were proportionally more taste-responsive cells in obese rats, suggesting potential compensatory mechanisms for diminished taste responses [14]. In addition to long-term high-energy diet exposure, short-term exposure to various tastants during early life also altered neural responsiveness to sucrose in the gustatory insular cortex (GC) of mice. Weanlings were given repeated access to various tastants—sucrose, citric acid, Ensure, and salt—with each solution presented once per day in a rotating manner over 8 days (each tastant was presented twice). Compared to naïve mice, this multi-tastant exposure group exhibited fewer sucrose-responsive pyramidal excitatory neurons and a decreased excitation/inhibition balance in the GC network [61].

In summary, and strikingly similar to insects, rodents display marked, reciprocal taste adaptations to long-term exposure to dietary environments.

Changes in taste with dietary composition have also been reported in humans. Most notably, lower sodium intake increased the sensitivity of saltiness [50,51]. In contrast, individuals who ate higher sodium had difficulties in identifying salt concentrations correctly [52], while those with severe obesity who ate a Mediterranean diet for 4 weeks showed a reduced salty threshold (indicating higher sensitivity to salt) but no change in the sweet threshold [55].

A similar trend is seen with sugar levels, where a decrease in the total carbohydrate intake over several months increased sweet sensitivity [38], while one month of soft drink supplementation was associated with lower sweet sensitivity at higher concentrations [39] and changes in sweet preference after sugary drink intake; no changes were observed in responses to salt. Moreover, switching from sugar-sweetened beverages (SSB) to either artificially sweetened (ASB) or unsweetened beverages (USB) over 12 months produced distinct effects on taste perception. The sweetness threshold and preferred concentration decreased in the USB group, while only the preferred concentration decreased in the ASB group [62]. A similar pattern was observed in participants who replaced SSB with water for 12 weeks, showing decreased liking for high sucrose concentrations [63]. Interestingly, blocking sweet taste sensation resulted in an increased desire for sweet foods [64]. Consistent with this, participants with higher sweet taste sensitivity consumed less total energy, carbohydrates, and sugars in their diets, while those with higher hedonic liking for sweetness tended to consume more total energy and sugars [65].

However, Mah et al. (2024) demonstrated that the relationship between reduced sugar intake and taste perception is more complex. Among habitual full-sugar sweetened beverage (FSS) consumers in the U.S., liking for low-sweetness beverages decreased in the control group (who continued FSS consumption for 6 months), but was maintained in both the stepwise (StepR) and direct (DirR) sweetness reduction groups over 6 months with no change in perceived sweetness intensity. In contrast, among habitual low-calorie sweetened beverage consumers in the U.S., sweetness intensity for high concentration of sucrose increased in the DirR group. Notably, no significant changes in liking or perceived intensity were observed in the Mexican cohorts, regardless of reduction strategy or baseline beverage habits [66].

Low-fat diet also influence sensory responses similarly, with low-fat diets increasing fat taste sensitivity in lean and obese individuals and high-fat diets reducing fat sensitivity in only lean individuals after 4 weeks [46,48]. Finally, an increase in the intake of the savory Monosodium glutamate resulted in a decrease in umami sensation and preference [53].

Thus, in humans, there is growing evidence that diet can influence taste perception, particularly in the context of chronic dietary patterns. However, the strength and consistency of this effect appear to vary by taste—being more robust for salt than for sweet taste. As highlighted in the review by Appleton et al. (2018) [67], findings on sweet taste are more mixed, and further work is needed to clarify these relationships.

How these changes affect eating and health is less clear but critically important to define. Notably, many studies in humans have looked at the relationship between body mass index and taste sensitivity and intensity; although a few found no effect, and others a positive correlation [13,68], most have reported a generally inverse relationship between the two, consistent with the findings of rodent and fly studies (reviewed in Bartoshuk et al. (2006) [69] and May and Dus (2021) [3]). However, it is important to note that in these models, diet had clear effects independent of weight gain.

## 4. Uncovering the Mechanisms of Diet-Induced Taste Plasticity

Much remains to be understood about the molecular and neural mechanisms underlying taste plasticity. Research using animal models is illuminating several key aspects, from metabolic signaling to inflammation.

Vaziri et al. (2020) used cell-specific gene expression measurements in the fly sweet-sensing neurons to show that a high-sugar diet decreased the mRNA abundance of 80% of expressed genes, with a particular reduction in synaptic genes [41]. This silencing was mediated by the conserved epigenetic repressor Polycomb Repressive Complex 2.1 (PRC2.1). Notably, 47% of the genes repressed by PRC2.1 under a sugar diet remained downregulated even after reverting to a control diet for seven days. A subsequent study linked increased PRC2.1 activity to the metabolic sensor O-linked *N*-Acetylglucosamine Transferase (OGT), whose activity rises with higher dietary sugar due to increased flux through the Hexosamine Biosynthesis Pathway [40]. While this metabolic–epigenetic (nutrigenomic) signaling drove taste adaptation prescriptively, the authors also found that it integrated directly with the activity of the neurons through the MAPK/ERK pathway [40]. These findings highlight a link between metabolic changes and neuronal signaling.

Expanding this link between metabolism and neural signaling, Wang et al. (2020) linked dietary modulation of sweet taste sensitivity in fruit flies to dopamine signaling and the Peroxisome Proliferator-Activated Receptor Gamma Coactivator 1-alpha (PGC1α) pathway [42]. This pathway modulates mitochondrial function and energy metabolism within sweet-taste neurons. Expanding on the role of dopamine, Ganguly et al. (2021) demonstrated that dopamine neuromodulation via the type 2-like receptor (Dop2R) enhances sweet sensitivity to sugar while simultaneously reducing sensitivity to amino acids under sugar-reduced/protein-enriched diets [54].

In addition to neural and metabolic pathways, endocrine signaling also contributes to the regulation of taste sensitivity. Notably, reduced sweet sensitivity correlated with the downregulation of *Drosophila insulin-like peptide 5 (Dilp5)* in response to a high-sugar/low-protein diet. Wang et al. (2016) further highlighted the role of insulin signaling in taste plasticity [37]—an intriguing finding, given that hormones such as leptin and insulin are also thought to influence the taste system in rodents (reviewed in May and Dus (2021) [3]). This area of research has been a significant focus of recent studies because of the rapid adoption of Glucagon-like peptide-1 (GLP-1) receptor agonists, such as semaglutide (Ozempic) for diabetes and obesity. This hormone has been increasingly recognized as influencing taste sensation [70,71,72,73]. GLP-1 is a peptide that stimulates the release of insulin in hyperglycemic states and reduces food intake via central and peripheral mechanisms [71,74,75,76,77]. Diets high in fiber or low in carbohydrates increase circulating GLP-1 plasma levels [78,79], while diets high in fat reduce circulating GLP-1 plasma levels [80,81]. In the peripheral taste system, GLP-1 is produced in taste cells in the circumvallate papillae that co-express T1R3, a subunit of both the sweet and umami taste receptors [72,82]. GLP-1 acts in the periphery to modulate sweet and umami taste sensitivity [71]. By enhancing GLP-1 receptor activation in the periphery, GLP-1 analogs may alter the functional responsiveness of taste cells, thus impacting taste sensation. Indeed, treatment with the GLP-1 analog liraglutide for 12 weeks resulted in significantly lower detection threshold of sweet and bitter tastes (indicating higher sensitivity to sweet and bitter) in type 2 diabetic patients [83]. Such alterations in taste sensation may lead to changes in dietary choices and intake patterns, which further influence taste plasticity.

Beyond endocrine mechanisms, inflammation due to diet-induced obesity also affects the taste system in rodents [84]. Obese mice fed a high-fat diet for eight weeks exhibited a large increase in relative gene expression of tumor necrosis factor-alpha (TNF-α), a primary marker of the innate immune response, in the tongue epithelial tissues containing taste buds; this rise in inflammatory markers coincided with reduced taste buds in circumvallate papillae [84]; the effect of obesity on the fungiform papillae was also observed in rats [15] and young males along with a deficit in salty and sweet taste [68,85]. However, a high dietary sugar, unlike fat, does not affect the number and distribution of taste papilla [43,44] but did decrease the number of type II PLCβ2+ taste receptor cells [44], although this effect disappeared when the sugar was removed from the rat diet. Interestingly, a decrease in α-gustducin and PLCβ2 mRNAs has been observed in the tongues of mice fed a chronic high-fat diet [20], and the sweet taste receptor T1R3 was significantly decreased in rats on a high-fat diet [86].

Rather specific changes in receptor expression have also been observed with chronic consumption of tastants for those receptors. Consumption of a high-fat diet resulted in a decrease in fat receptor CD36 expression in circumvallate taste buds in mice [12,47]. Furthermore, dietary fat intake also modulates fatty acid taste receptors in humans. Chronic consumption of a low-fat diet showed to upregulate fat receptor FFAR4 expression in fungiform papillae, which is associated with increased fat taste sensitivity [49]. Receptor mRNA expression was also lower after exposure to appetitive, but not to aversive tastants for four weeks, specifically, lower mRNA abundance of the umami receptor (Tas1R1), sweet receptor (Tas1R2), epithelial sodium channel (ENaC) for salt, and polycystic kidney disease 2-like 1 protein (PKD2L1), which is considered a marker of sour-sensitive taste cells [87], in mice treated with MSG, saccharin, NaCl, and citric acid, respectively, but no changes in bitter receptor (Tas2R5) with quinine treatment [56]. Notably, early-life exposure to low-calorie sweeteners also influences the taste system. Juvenile rats given chronic access to acesulfame potassium exhibited a significant reduction in the sweet taste receptors Tas1R2 and Tas1R3 expression in the circumvallate papillae, leading to decreased responsiveness to sugars [45,88]. Similar receptor-based adaptations occur in fruit flies as well. Zhang et al. (2013) found the fly bitter taste adaptation operated through direct modulation of receptor proteins. The exposure to a camphor-supplemented diet downregulates the transient receptor potential-like (TRPL) cation channel in gustatory neurons, thereby reducing camphor aversion [8]. This effect was mediated by protein turnover via the E3 ubiquitin ligase Ube3a, with TRPL transcription remaining unchanged.

**Figure 1:**
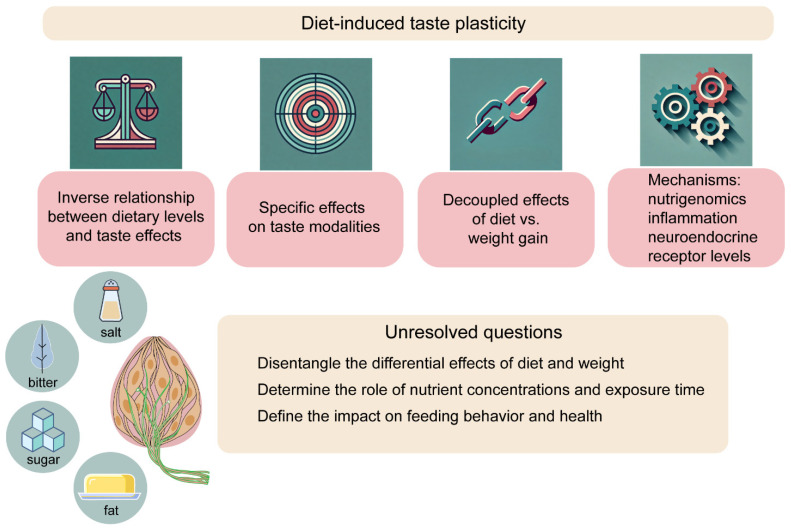
Evolving principles and unresolved questions in diet-induced taste plasticity.

Overall, while significant gaps remain in understanding the mechanisms of diet-induced taste plasticity, recent studies highlight metabolic, signaling, and gene expression changes driven by specific nutrient conditions, particularly in humans and models exposed to high-fat and high-sugar diets. Among the unresolved questions, the most pressing include disentangling the differential effects of diet and weight, the role of nutrient concentrations and exposure durations, and the impact of taste adaptations on feeding behavior and overall health (Figure 1). On a broader scale, the evolution of sensory adaptations, such as those observed in the arms race between plants and insects, is an intriguing area of exploration with potential applications in pest control.

## 5. Conclusions

Diet-induced taste plasticity is a unique neurobiological process that highlights the sensory system’s remarkable adaptability while presenting feasible opportunities for sustainable public health interventions. Although much remains to be uncovered about the drivers and consequences of this plasticity, recent studies have begun to define some fundamental principles. First, there is generally an inverse correlation between the levels of a nutrient in the diet and its effect on taste sensation; second, the effects are often, but not universally specific to the taste modality linked to the nutrient; third, there are different, decoupled effects coming from diet exposure vs. obesity; and finally, the mechanisms span from protein and gene regulation to metabolic signaling, inflammation, and neuroendocrine pathways (Figure 1). What is evident is that taste, and its adaptability, lies at a critical intersection between nutrition and health. Investigating its mechanisms will deepen our understanding of nutrient-sensing processes in the mouth, brain, and body. Finally, characterizing this phenomenon will be a robust foundation for designing foods and healthy eating strategies that enhance and safeguard well-being.

## Figures and Tables

**Table 1:** Summary of notable findings on the diet-induced taste.

Diet	Organism [Ref]	Effect on Taste Sensation	Taste System Alteration/Underlying Mechanisms
**Bitter**			
Strychnine	Caterpillars of the moth [6]	Diminished responses to strychnine	Unidentified
Strychnine	Larvae of white butterfly [7]	Diminished responses to bitter compounds	Unidentified
Caffeine	Caterpillars of the moth [9,10]	Diminished responses to caffeine	Unidentified
Camphor	Fruit flies [8]	Diminished responses to camphor	Downregulated TRPL mediated via Ube3a in sweet neurons
**Sweet**			
Non-nutritive sweetener	Fruit flies [37]	Increased responses to low concentrations of sucrose	Unidentified
Low sugar	Humans [38]	Increased perceived sweet intensity	Unidentified
Soft drink supplementation	Humans [39]	Reduced perceived sweet intensity	Unidentified
High sugar	Fruit flies [19,40,41]	Desensitized responses to sucrose	Epigenetic modulation mediated by PRC2.1/OGT MAPK/ERK signaling pathway
Sorbitol	Fruit flies [42]	Increased sensitivity to sucrose	Dopamine signaling via DopR1 and PGC1α pathway
High sugar	Rats [43,44]	Attenuated responses to sucrose	Decreased PLCβ2+ type II taste cells in fungiform papillae
Low-calorie sweetener	Rats [45]	Unidentified	Reduction in Tas1R2 and Tas1R3 in circumvallate papilla
**Fat**			
High fat	Humans [46]	Increased fat (C18:1) taste threshold	Unidentified
High fat	Mice [20]	Reduced responses of taste cells to sucralose and denatonium	Reduction in mRNA of α-gustducin and PLCβ2 in taste buds
High fat	Mice [47]	Unidentified	Reduced mRNA of CD36 in circumvallate papillae
Low fat	Humans [46,48,49]	Decreased fat (C18:1) taste threshold	Upregulated mRNA of FFAR4 in fungiform papillae
**Salt**			
Low salt	Humans [50,51]	Reduced salty threshold	Unidentified
High salt	Humans [52]	Difficulty in discriminating salt concentration	Unidentified
**Umami**			
Monosodium glutamate	Humans [53]	Reduced perceived umami taste intensity	Unidentified
**Protein**			
Protein restricted	Fruit flies [42]	Increased sensitivity to sucrose	Dopamine signaling via DopR1 and PGC1α pathway
**Other**			
Sugar-enriched/Protein-depleted	Fruit flies [54]	Decreased responses to sugars/ Increased responses to amino acids	Downregulation of Dilp5
Sugar-reduced/Protein-enriched	Fruit flies [54]	Increased responses to sugars	Dopamine neuromodulation via Dop2R
Mediterranean	Human [55]	Reduced salty threshold	Unidentified
Appetitive taste solutions	Mice [56]	Unidentified	Decreased mRNA for Tas1R1, Tas1R2, or ENaC
Aversive taste solutions	Mice [56]	Unidentified	Increased mRNA for Tas2R5 or PKD2L1

## Data Availability

The raw data supporting the conclusions of this article will be made available by the authors on request due to (specify the reason for the restriction, e.g., privacy, legal or ethical reasons).
